# Syphilis, Hepatitis, and Pancreatitis: Is the Uncommon Becoming Common in the HIV^+^ Patient?

**DOI:** 10.1155/2013/293823

**Published:** 2013-12-09

**Authors:** B. A. da Silva, T. S. Soi, D. Cameron, A. C. Karikkineth, R. B. Williams

**Affiliations:** Department of Internal Medicine, MedStar Harbor Hospital, 3001 South Hanover Street, Baltimore, MD 21225, USA

## Abstract

*Background*. Coinfection with human immunodeficiency virus (HIV) and syphilis has been occurring at increasing rates, with the greatest increases being seen among men who have sex with men. Secondary syphilis rarely presents with liver disease, and the diagnosis may be overlooked in favor of more common causes of liver injury in this setting, such as viral hepatitis, antiretroviral therapy, alcohol use, and opportunistic infections. *Case Presentation*. We describe a 43-year-old patient with HIV who presented with symptoms suggesting acute pancreatitis. Investigation led to a diagnosis of hepatitis and pancreatitis, both attributed to syphilis. *Conclusion*. Syphilis should be included as part of the initial diagnosis among patients with HIV presenting with abnormal liver and pancreatic enzymes.

## 1. Introduction

**Figure 1:**
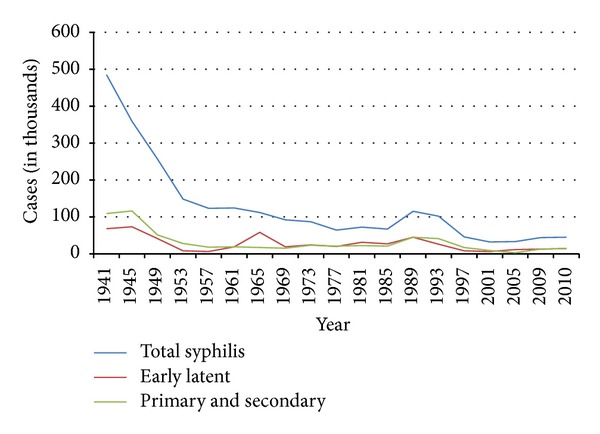
Syphilis—Reported Cases by Stage of Infection, United States, 1941–2010. Centers for Disease Control and Prevention. *STD Trends in the United States 2012*. Atlanta: U.S. Department of Health and Human Services; 2012.

**Figure 2:**
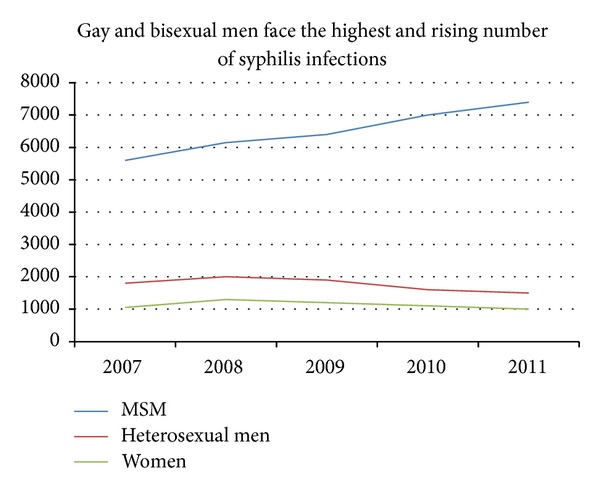
Syphilis—Reported Cases by Stage of Infection, United States, 1941–2011. Centers for Disease Control and Prevention. *STD Trends in the United States 2012*. Atlanta: U.S. Department of Health and Human Services; 2012.

The diagnosis of human immunodeficiency virus (HIV) begins with clinical suspicion based on signs and symptoms, physical exam findings, and social risk factors. Coinfection with HIV and syphilis has been occurring at increasing rates, with the greatest increases being seen among men who have sex with men (MSM). While secondary syphilis rarely presents with liver disease, a diagnosis of syphilis in a patient with HIV should be considered, particularly when there is evidence of liver and pancreatic inflammation. Infection rate of *Treponema pallidum *declined by 89.7% during the years 1990–2000; however, an increase occurred between 2001 and 2009; MSM are mainly responsible for this increase [1] (Figure 1). Syphilis is associated with increasing rates of HIV coinfection; in the year 2011, 40% of cases were coinfected with HIV (Figure 2) [2].

All at-risk patients should be screened for HIV, especially if being evaluated for any sexually transmitted infections (STIs). Coinfection of HIV with an STI may amplify disease and infectivity, but mechanisms and magnitude are still largely unknown. A 2007 retrospective study composed of 282 HIV^+^ men who were also diagnosed with syphilis demonstrated an average increase in HIV viral load of 54,000 copies/mL. A similar increase of viral load levels was noted among men on antiretroviral therapy. [3] Several other reports support these findings.

Interestingly, CD4^+^ counts have been shown to dramatically decline during an acute syphilis coinfection [3–5]. Screening for syphilis in high-risk groups is not only justified but also necessary given the increased rates of coinfection and detrimental effects on HIV status.

Few data are available on the prevalence of syphilitic hepatitis in HIV^+^ patients. We argue that rare presentations of syphilis within healthy populations may become more common presentation in HIV^+^ cases. One of the first studies to investigate the prevalence of syphilitic hepatitis in HIV^+^ populations found that 19.3% of patients within their study population had abnormal liver enzymes consistent with syphilitic hepatitis [6]. It is suggested that 2.7% to 10% of HIV^+^ individuals with abnormal liver enzymes will be diagnosed with syphilitic hepatitis [7, 8]. It is becoming recommended and more well understood that high-risk patients with liver enzyme abnormalities should be screened for syphilis. Follow-up lab work should be obtained until liver enzymes return to normal levels.

To date there have been no studies or data gathered on prevalence or incidence of syphilis affecting the pancreas. Extremely few case reports document a similar or suspected finding. Herein we describe what we believe to be syphilis leading to pancreatitis and hepatitis in an HIV^+^ patient.

## 2. Case Presentation

A 43-year-old homosexual and HIV^+^ gentleman presented to the Harbor Hospital Emergency Department with one week of nausea, vomiting, and severe abdominal pain. The pain was diffuse, intermittent, and dull but was most prominent at the epigastrium and right upper quadrant. It became more painful over the previous week. The patient had several episodes of nonbloody vomiting and oral intake made his pain and nausea worse. He denied diarrhea, change of bowel habits, or fever and chills. The patient did endorse a five-pound weight loss over the last month. One month earlier, he was seen at another institution for a similar complaint and was found to have sigmoid thickening and retroperitoneal lymphadenopathy. He received no followup.

His HIV treatment consisted of efavirenz/tenofovir/emtricitabine and he stated compliance for the last several years. Blood work from several months earlier revealed CD4^+^ T-cell count of 534 cell/mm^3^ and viral load of 37 copies/mL. Besides gastroesophageal reflux, he had no other medical history and took no other medications. He had a 30-pack-year history of tobacco use and denied any illegal drug abuse. The patient admitted to consuming 3 glasses of wine at a party a couple of days earlier but normally he does not consume alcohol. Intimate sexual history was not obtained other than homosexual preference. Upon admission, the patient's antiretroviral medications were continued.

**Figure 3:**
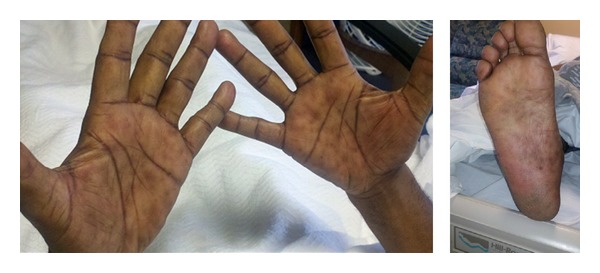
Macular rash on bilateral palms and right plantar surface. Used with permission: Department of Internal Medicine, MedStar Harbor Hospital.

On physical exam the patient was afebrile and normotensive. His abdomen was soft, nondistended, and diffusely tender. He was particularly tender at the right upper quadrant and demonstrated voluntary guarding. He had a macular, darkly pigmented rash on the palms and soles, as well as bilateral nontender inguinal lymphadenopathy (Figure 3). Genitourinary and rectal exams were unremarkable. The complete blood cell count was within acceptable limits, and renal function was unremarkable. CD4^+^ T-cell count was 394 cells/mm^3^. Further workup revealed alkaline phosphatase of 712 IU/L (normal 30–115 IU/L), AST of 262 IU/L (normal 9–40 IU/L), ALT of 481 IU/L (normal 7–55 IU/L), a total bilirubin of 1.7 mg/dL (normal 0.0-1.0 mg/dL), and a lipase of 1396 U/L (normal 12–70 U/L). Serological tests were negative for acute or chronic viral hepatitis and the patient demonstrated immunity to hepatitis B. Lipid panel was insignificant.

The patient was thought to have acute pancreatitis secondary to suspected gallstones. Abdominal X-ray revealed no significant findings. Abdominal ultrasound revealed a contracted gallbladder, but no stones were visible. Magnetic resonance cholangiopancreatography (MRCP) was negative. Computed tomography (CT) of the abdomen revealed bilateral lower lobe infiltrates and a follow-up CT of the chest revealed multilobar infiltrates. The patient was started on vancomycin, azithromycin, and piperacillin/tazobactam. Several days elapsed with similarly elevated liver and pancreatic enzymes. An RPR was ordered and returned weakly positive at 1 : 8 and a *T. pallidum* antibody test returned positive. Treatment was initiated with intramuscular benzathine penicillin G (2.4 million units) given weekly for three weeks. Over the next two weeks liver and pancreatic enzyme levels declined. His antiretroviral medications were never discontinued. After antibiotic completion, followup with the patient found him feeling well. A repeat alkaline phosphatase was 198 IU/L, AST 31 IU/L, ALT 45 IU/L, and lipase 121 U/L.

## 3. Discussion

Hepatitis represents a unique and rare manifestation of secondary syphilis. A diagnosis of syphilitic hepatitis is best suggested in the setting of abnormal liver enzymes, serological evidence for syphilis, exclusion of alternative causes, and improvement in liver function following antimicrobial therapy. It is important to note that HIV^+^ patients typically have very high RPR titer levels [9]. It is becoming more well known and accepted that syphilis can lead to hepatitis, but research on syphilitic pancreatitis is severely lacking.

There are scarce case reports available linking syphilis to pancreatitis, but the data does exist to suggest this association. Syphilitic pancreatitis is an extremely rare and poorly understood complication of syphilis [10–14]. Patients on antiretroviral therapy can have elevations of liver and lipase enzymes secondary to medication induced hepatitis and pancreatitis. It is thus recommended that these enzymes be followed for acute changes.

Patients who continue to receive HAART and have decreasing LFTs and lipase levels can have medication induced inflammation ruled out. As in our patient, declining hepatic and pancreatic enzymes after completing penicillin therapy truly suggests syphilis infection as the inciting event.

## 4. Conclusion

In HIV^+^ individuals presenting with abnormal liver and pancreatic enzymes, syphilis should be included as an important diagnosis. Both hepatitis and pancreatitis should be considered.
